# The Role of Mitogen-Activated Protein (MAP) Kinase Signaling Components in the Fungal Development, Stress Response and Virulence of the Fungal Cereal Pathogen *Bipolaris sorokiniana*

**DOI:** 10.1371/journal.pone.0128291

**Published:** 2015-05-26

**Authors:** Yueqiang Leng, Shaobin Zhong

**Affiliations:** Department of Plant Pathology, North Dakota State University, Fargo, North Dakota, United States of America; Zhejiang University, CHINA

## Abstract

Mitogen-activated protein kinases (MAPKs) have been demonstrated to be involved in fungal development, sexual reproduction, pathogenicity and/or virulence in many filamentous plant pathogenic fungi, but genes for MAPKs in the fungal cereal pathogen *Bipolaris sorokiniana* have not been characterized. In this study, orthologues of three MAPK genes (*CsSLT2*, *CsHOG1* and *CsFUS3*) and one MAPK kinase kinase (MAPKKK) gene (*CsSTE11)* were identified in the whole genome sequence of the *B*. *sorokiniana* isolate ND90Pr, and knockout mutants were generated for each of them. The *∆Csfus3* and *∆Csste11* mutants were defective in conidiation and formation of appressoria-like structures, showed hypersensitivity to oxidative stress and lost pathogenicity on non-wounded leaves of barley cv. Bowman. When inoculated on wounded leaves of Bowman, the *∆Csfus3* and *∆Csste11* mutants were reduced in virulence compared to the wild type. No morphological changes were observed in the *∆Cshog1* mutants in comparison with the wild type; however, they were slightly reduced in growth under oxidative stress and were hypersensitive to hyperosmotic stress. The *∆Cshog1* mutants formed normal appressoria-like structures but were reduced in virulence when inoculated on Bowman leaves. The *∆Csslt2* mutants produced more vegetative hyphae, had lighter pigmentation, were more sensitive to cell wall degrading enzymes, and were reduced in virulence on Bowman leaves, although they formed normal appressoria like the wild type. Root infection assays indicated that the *∆Cshog1* and *∆Csslt2* mutants were able to infect barley roots while the *∆Csfus3* and *∆Csste11* failed to cause any symptoms. However, no significant difference in virulence was observed for *∆Cshog1* mutants while *∆Csslt2* mutants showed significantly reduced virulence on barley roots in comparison with the wild type. Our results indicated that all of these MAPK and MAPKKK genes are involved in the regulation of fungal development under normal and stress conditions and required for full virulence on barley plants.

## Introduction

Mitogen-activated protein kinases (MAPKs) have been demonstrated to regulate specialized responses in eukaryotic organisms to environmental signals and molecules from other organisms [1–4]. Functions of MAPK pathways were first studied in the budding yeast, *Saccharomyces cerevisiae*, which serves as a model for other filamentous fungi [2, 5]. In *S*. *cerevisiae*, the pheromone response pathway is well characterized, where the mating process is regulated by the Ste11-Ste7-Fus3/Kss1 cascade. Several elements of this pathway are found to be involved in filamentous growth of fungi [2]. One of these components, Ste11, is also used by the high osmolarity glycerol (HOG) pathway, which is required for the fungal growth under hyperosmotic conditions [2, 6]. Another MAPK pathway of *S*. *cerevisiae* is the Pkc1-Slt2 pathway that mainly regulates the cell wall integrity and promotes cell wall biosynthesis [2]. In filamentous plant pathogenic fungi, MAPK pathways are found to be required for fungal development and full virulence on hosts [3]. Three classes of MAPKs have been identified in ascomycetes. The first class of MAPKs is represented in *Magnaporthe grisea* by *PMK1* (Pathogenicity MAP Kinase 1), which is orthologous to *FUS3/KSS1* of *S*. *cerevisiae* [1, 7]. This MAPK is required for formation of appressoria and infectious hyphae [1, 7]. The second class of MAPKs is represented in *M*. *grisea* by *OSM1*, an ortholog of *HOG1* in the budding yeast. The deletion mutant of *OSM1* (*∆osm1*) shows normal virulence on rice but is sensitive to osmotic stress [8]. The third class of MAPKs is represented in *M*. *grisea* by *MPS1*, which is orthologous to *STL2* in *S*. *cerevisiae*. This MAPK is required for cell wall integrity and appressorial penetration. The *∆mps1* mutants of *M*. *grisea* show weakened cell walls and are unable to infect rice [9]. In the southern corn leaf blight fungus, *Cochliobolus heterostrophus*, three MAPKs genes (*HOG1*, *MPS1* and *CHK1*) have been identified and characterized [3]. The *∆chk1* and *∆mps1* mutants are reduced in virulence, conidium formation and pigmentation compared to the wild type [3]. Although the *C*. *heterostrophus MPS1* is 83.6% identical to the *MPS1* of *M*. *grisea*, the function is different in the two fungi [3, 9]. In *M*. *grisea*, *∆mps1* mutants form appressoria but are unable to penetrate and thus lose pathogenicity [9]. In contrast, the *∆mps1* mutants of *C*. *heterostrophus* form normal-looking appressoria and are able to penetrate plant cells although they are reduced in virulence compared to the wild type [3]. In *C*. *heterostrophus*, *HOG1* was found to be involved in resistance to hyperosmotic and oxidative stresses, formation of appressoria and virulence [3]. *Chste11* (an ortholog of *STE11* in *S*. *cerevisiae*) was also found to be essential for sexual or asexual development, appressorial formation and pathogenicity in *C*. *heterostrophus* [6].

Although MAPK pathways are highly conserved and their components have been studied in a number of fungi [2, 3], none of the MAPK genes has been functionally characterized in *Bipolaris sorokiniana*, the causal agent of spot blotch, common root rot and kernel blight in barley and wheat [10, 11]. In this study, we generated knockout mutants of the gene orthologues for four MAPK signaling components (*CsFUS3*, *CsSLT2*, *CsHOG1* and *CsSTE11*) in *B*. *sorokiniana*, and demonstrated their roles in fungal development and virulence on leaves and roots of barley plants.

## Materials and Methods

### Fungal isolates, growth media and assays for stress sensitivity and conidial production

The *B*. *sorokiniana* isolate ND90Pr was used as the recipient for all transformation experiments. ND90Pr is classified as a pathotype 2 isolate, which is highly virulent on barley cv. Bowman, but exhibits low virulence on the other two barley lines (ND 5883 and ND B112) [12]. The conditions and media described by Leng et al. [13] were used for culturing isolates of *B*. *sorokiniana*. To test the sensitivity of fungal isolates to different stresses, a small mycelial plug (2×2 mm^2^) from a three days old fresh fungal culture was inoculated onto the center of agar plates supplemented with individual reagents. Sensitivity to hydrogen peroxide (H_2_O_2_) was determined on PDA supplemented with 10mM H_2_O_2_. The plates were incubated at 25°C for six days in the dark, and then the diameters of fungal colonies were measured and photographed. Sensitivities to other stresses were determined on PDA plates supplemented with 1M KCl or 1.5 M sorbitol. The plates were grown in a cycle of 14 h of light and 10 h of darkness for six days before the diameters of fungal colonies were measured and photographs taken.

To compare conidial productivity of different *B*. *sorokiniana* strains, small mycelial plugs (2×2 mm^2^) from three-day old fungal cultures of each strain were inoculated on the centers of minimal medium [14] plates and allowed to grow for six days in a cycle of 14 h of light and 10 h of darkness. Conidia were harvested by adding 10 ml of distilled water to the plate and scraping the agar surface with a rubber spatula, followed by adding another 10 ml of distilled water to wash off all conidia on the plate, and then filtration through two layers of cheesecloth to remove mycelial fragments. A hemocytometer was used to count conidia and the average number of conidia from each strain was calculated from three replicate plates.

### Identification of MAPK gene orthologues in *B*. *sorokiniana*

The orthologues of *FUS3*, *SLT2*, *HOG1* and *STE11* in *B*. *sorokiniana* were identified by BLAST search against a draft genome sequence of the *B*. *sorokiniana* strain ND90Pr [15] using the sequences of MAP Kinase genes reported in *C*. *heterostrophus* [3, 5, 6, 16].

### Generation of gene knockout mutants and complemented strains

**Fig 1:**
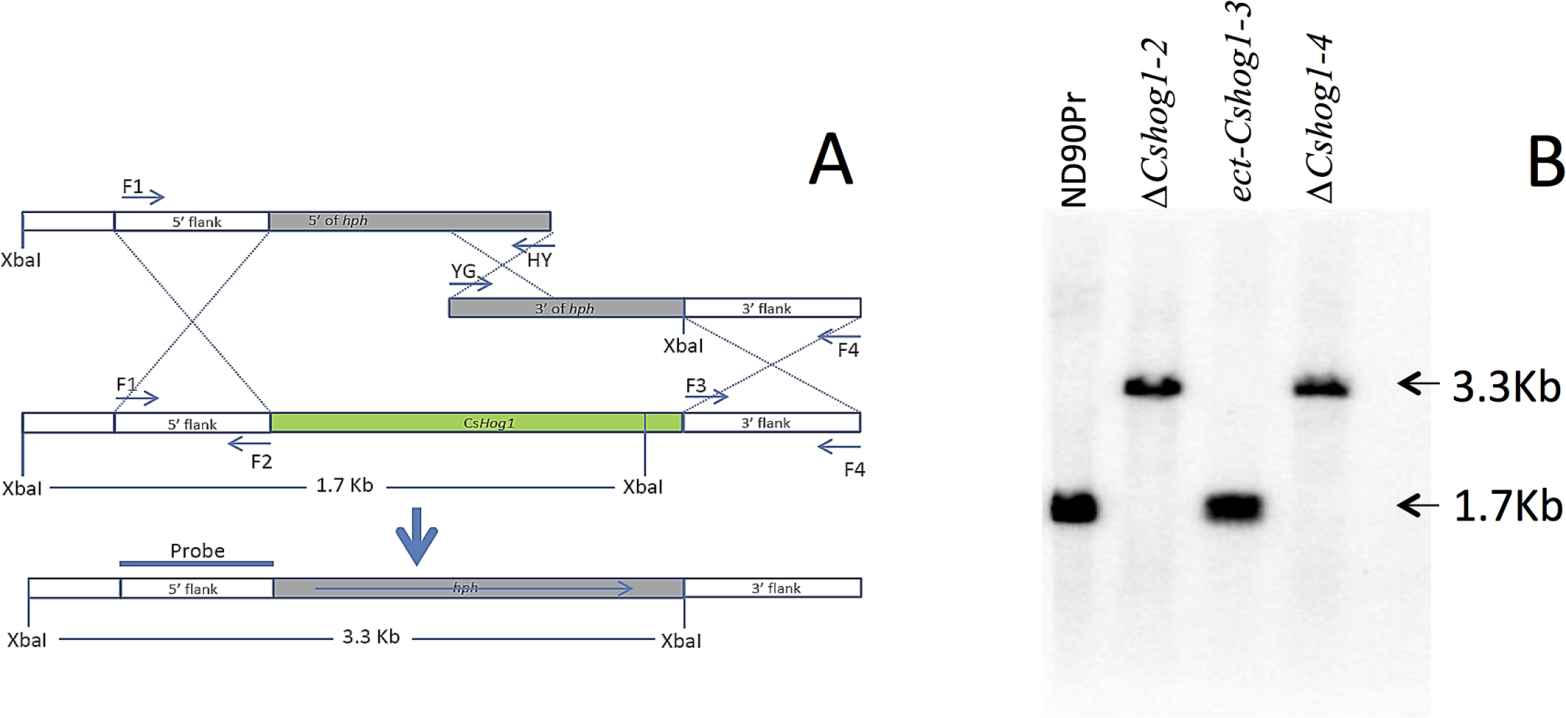
Generation of ∆*Cshog1* strains of *Bipolaris sorokiniana*. A, a diagram showing replacement of the *CsHOG1* gene by a 2.6 kb fragment carrying the *E*. *coli* hygromycin phosphotransferase gene (*hph*) using the split-marker system [17]. B, Southern hybridization of *Xba* I-digested genomic DNA from the wild type and ∆*Cshog1* strains using probe amplified with primers CsHOG1-F1+CsHOG1-F2. The 1.7 kb fragment in the wild type strain (ND90Pr) was replaced by the 3.3 kb fragment in the ∆*Cshog1* strains (∆*Cshog1*-2 and ∆*Cshog1*-4). *ect*-*Cshog1*-3 is an ectopic transformant.

The split marker system [17] was used for gene deletion or replacement (Fig 1 and S1–S3 Figs). The 5' and 3’ flanking sequences of the target gene were amplified using the specific primers designed for each gene (Table 1). The PCR reaction (50 μl) contained 30 ng of fungal genomic DNA, 5 pmol of each primer, 0.2 mM of each deoxynucleoside triphosphate (dNTP), 1 × reaction buffer [20 mM Tris-Cl, 2.0 mM MgSO_4_, 50mM KCl, 10 mM (NH_4_)_2_SO_4_, 0.1% Triton X-100, pH 8.8] and 2.5 U of Taq DNA polymerase (New England Biolabs, Ipswich, MA, USA), and was performed using a Mastercycler PTC-100 (MJ Research, Ramsey, MN, USA). The thermal cycling conditions were as follows: initial denaturation (95°C, 2 min), followed by 35 cycles of denaturation (94°C, 30 s), annealing (58°C, 30 s) and extension (72°C, 1 min), and then one final cycle of extension (72°C, 10 min). The overlapping fragments of hygromycin resistance gene cassette were amplified from pDAN-*ToxA* [18] using the M13F/HY and M13R/YG primers, respectively (Table 1) using the PCR conditions described above except for the annealing temperature (60°C). To generate the 5’ construct, PCR products amplified by F1/F2 and M13F/HY were used as templates, and primers F1/HY were used in the fusion PCR. To generate the 3’ construct, PCR products amplified by F3/F4 and M13R/YG were used as the templates and primers were F4 and YG in the fusion PCR. The 3’ and 5’ constructs generated were mixed, purified by ethanol precipitate, and then used for transformation.

**Table 1:** Primers used in this study.

Primer name	Primer sequence (5’–3’)*
M13F	GACGTTGTAAAACGACGGCCAGTG
M13R	CACAGGAAACAGCTATGACCATGA
HY	GGATGCCTCCGCTCGAAGTA
YG	CGTTGCAAGACCTGCCTGAA
CsFUS3-F1	AGGAAGCGGCAGCTCCAGGAA
CsFUS3-F2	CACTGGCCGTCGTTTTACAACGTCGCTCTTGTGTGCGAATGAC
CsFUS3-F3	TCATGGTCATAGCTGTTTCCTGTGGCTTCGACCGAGGACAACT
CsFUS3-F4	GCTCAAGGTATGGGTGCTTGA
CsFUS3-com-F	CGGCGAGATGTCAGTTTGTA
CsFUS3-com-R	CTGCAGATAAGCTGCGAAAA
CsHOG1-F1	CCATACCCTGGTGCAAACTC
CsHOG1-F2	CACTGGCCGTCGTTTTACAACGTCCGTACCAAACGAGACCGAAT
CsHOG1-F3	TCATGGTCATAGCTGTTTCCTGTGCCAGTCGACACCTGGAAGAT
CsHOG1-F4	GAGCAGATGCATCACCAAGA
CsSTE11-F1	ACCACACTACACACCCGTCA
CsSTE11-F2	CACTGGCCGTCGTTTTACAACGTCCCAGTCACCaACCTTGTCCT
CsSTE11-F3	TCATGGTCATAGCTGTTTCCTGTGATTTGGTCACTTGGCTGCTT
CsSTE11-F4	ACAGAATCATCTCGCGCATA
CsSTE11-F5	GCGTTGCCTCTAGCTTGAAA
CsSTE11-F6	GGGCACCCTTCATGTATTTG
CsSLT2-F0	GGTCTGAGCAGCATGACCA
CsSLT2-F1	AGCCTTGGGCCATATCTTCT
CsSLT2-F2	CACTGGCCGTCGTTTTACAACGTCCTTGCTGAAGACGTTGGTGA
CsSLT2-F3	TCATGGTCATAGCTGTTTCCTGTGTCAGGTCCCGATACCCAATA
CsSLT2-F4	CTGATTTCACGTCTGGCTCA
CsSLT2-com-F	ACGGAGACTGATGAGGCACT
CsSLT2-com-R	CTGGGTGTTGGGATACTGGT

*Underlined sequences are complementary to M13F and M13R sequences, respectively.

To generate the complemented strains, the coding regions of *CsFUS3* and *CsSLT2* and their upstream promoters (1764 bp and 1010 bp) were amplified using primer pairs CsFUS3-com-F+ CsFUS3-com-R and CsSLT2-com-F+ CsSLT2-com-R, respectively (Table 1). The PCR products were cloned into the vector pGEM-T Easy (Promega, Madison, WI, USA) according to the manufacturer’s protocol. The plasmid with the targeted gene insertion was mixed with the plasmid pBG418 carrying the gene for geneticin resistance [13] for co-transformation of a knockout mutant (*∆Csfus3* or *∆Csslt2*).

### Fungal transformation

Protoplast preparation was performed according to Zhong et al. [19] and transformation was carried out via the polyethylene glycol (PEG)-mediated method according to the procedure of Turgeon et al. [20] with some modifications. Flasks containing 250 ml of PDB medium were inoculated with 10^7^ conidia and incubated for 24 hours at room temperature (23–25°C) by shaking in an incubator shaker at 150 rpm. Fresh mycelia were harvested using two layers of miracloth and washed twice with sterile water and twice with the mycelium washing solution (0.7M KCl, 1.47g/L CaCl_2_). The mycelia then were resuspended in 40 ml of enzyme solution [400mg Lysing enzyme (Sigma-Aldrich, St. Louis, MO) and 200 mg Driselase (Sigma-Aldrich, St. Louis, MO) dissolved in 40 ml washing solution] and incubated for 3–5 hours at 30°C. Protoplasts were harvested by pouring the solution through four layers of miracloth and centrifugation at 3500g for 10 min. The pellet protoplasts were suspended in STC (Sorbitol, 1.2 M; Tris-HCl, 10 mM, pH 7.5; CaCl_2_, 10 mM): PEG [PEG, MW 3350 (Sigma-Aldrich, St. Louis, MO), 50 g/100 ml; Tris-HCl, 10 mM, pH 7.5; CaCl_2_, 10 mM] (4:1) and adjusted to a concentration of 2×10^8^/ml. Linearized plasmid DNA or purified PCR products [in a volume of less than 20 μl in STC: PEG (4:1)] were added to 100 μl protoplast suspension, mixed and incubated on ice for 20 min. Then, 100 μl, 300 μl and 600 μl PEG were added to the sample orderly and incubated for 20 min at room temperature. Finally, 1 ml, 3 ml and 4 ml STC were added to the sample orderly and mixed gently. Protoplasts were pelleted by centrifugation at 3500 g for 10 min, re-suspended in 1.6 ml RM (Sucrose, 1 M; Yeast extract, 0.1%; Tryptone, 0.1%) and incubated at room temperature for 2–4 hours by gently shaking at 75 rpm. The protoplast suspension then was mixed gently with 20 ml RMA (0.8% regeneration medium agar at 55°C containing appropriate antibiotics [hygromycin B (Roche Applied Science, Indianapolis, IN USA)] at 50 μg/ml. The mixture was poured into a Petri plate and incubated at 28°C. After 3–5 days, the transformants were transferred to fresh PDA or V8 PDA with appropriate antibiotics and incubated at room temperature. The transformants were purified by single spore isolation and hyphae tipping, and then stored on silica gels using the method of Windels et al. [21].

### Southern hybridization

Genomic DNA was isolated according to Zhong et al. [19] and digested with with *Xba* I or *Eco*R V. The digests were fractionated on a 0.8% agarose gel in 1×TAE (40 mM Tris, 20 mM acetic acid, and 1 mM EDTA, pH8.0) and transferred to Hybond N+ (Amersham Biosciences, Piscataway, NJ, USA). The probes used to detect the deletion of *CsHOG1*, *CsFUS3 and CsSTE11* were amplified with primer pairs indicated in Fig 1 and S1 and S2 Figs, respectively, and labeled with α-[32P]-dCTP using DNA Polymerase I (Promega, Madison, WI, USA). The hybridization and detection procedures were performed according to Zhong et al. [19]. To detect the deletion of *CsSLT2* in *B*. *sorokiniana*, the primer pair CsSLT2-F0/HY (Table 1) was used for PCR with DNA templates from the wild type and mutants (S3 Fig).

### Pathogenicity test on barley leaves

Pathogenicity tests were performed by spray inoculation with conidia suspension or point inoculation with mycelial plugs (2×2 mm^2^) on two-week-old seedlings of barley cv. Bowman. The spray inoculations were carried out according to Fetch and Steffenson [22] except that spore suspensions at 2×10^3^ conidia/ml were used. For point inoculation, fully-expanded second leaves (intact or wounded) of two-week-old barley (cv. Bowman) plants were inoculated with small mycelial plugs (2×2 mm^2^). For inoculation of intact barley leaves, individual mycelial plugs were placed on the middle portion of the leaves, separated at a distance of 2 cm. For inoculation of wounded leaves, individual mycelia plugs were placed at the wounded sites generated by a syringe needle (1CC). Inoculated plants were incubated in a humidity chamber for 18–24 hours, and then transferred into a growth chamber (20±2°C) and incubated for 4–7 days before disease ratings. The 1–9 rating scale of Fetch and Steffenson [22] was used to rate the spot blotch disease for spray inoculation experiments. Lesion sizes were measured when point inoculation was used.

### Pathogenicity test on barley seedling roots

Root infection was carried out according to the method described by Liljeroth et al. [23]. Briefly, seeds of barley cv. Bowman were allowed to germinate on wet filter paper in petri plates. When roots reached 2 mm long, the seeds were placed on a cellulose filter paper sheet (400×220 mm^2^) and another filter paper sheet with a plastic wrap was placed on top. The resulting stacks were rolled together and the lower end of the roll was placed in the flask with distilled water. Roots which grew to about 15 cm long were inoculated by placing mycelial plugs (2×2 mm^2^) on positions at a 5 cm distance from the base of the seedlings. The length of brown discoloration root lesions was measured at 9 days after inoculation. For each strain, 27 roots from 9 seeds were inoculated. All experiments were repeated twice.

### Microscopic examination for infection structure

To examine the infection structures of the wild type and the deletion mutants *in planta*, two-week old seedlings of barley cv. Bowman were inoculated with a spore suspension of 5×10^3^ conidia/ml prepared from each of the strains. At 24 hours after inoculation (HAI), leaf segments were sampled and subjected to treatments according to the method described by Koch and Slusarenko [24] with some modifications. Briefly, the infected leaf samples were cut into pieces and immersed in the trypan blue staining solution prepared by diluting the stock staining solution (0.02 g trypan blue, 10 g phenol, 10 ml glycerol, 10 ml lactic acid and 10 ml water) with 96% ethanol (1:2 v/v). The staining solution with samples was boiled in a water bath for one minute and left overnight at room temperature. The stained samples were subsequently destained by washing samples with chloral hydrate solution (100 g of chloral hydrate in 40 ml water) twice, and then examined under an Olympus BX51 microscope (Olympus, Center Valley, PA, USA) with the images recorded by a CCD camera (Diagnostic Instruments, Inc., Sterling Heights, MI, USA). The fungal structures and dead plant cells were stained blue by this method.

## Results

### Identification of MAPK and MAPKKK genes in *B*. *sorokiniana*

A BLAST search was done against the *B*. *sorokiniana* genome sequence [15] with the *HOG1*, *MPS1*, *CHK1*, *STE11* sequences of *C*. *hetrostrophus* [3, 5, 6, 16]. Orthologues of these genes were identified in *B*. *sorokiniana*, which were designated as *CsHOG1*, *CsSLT2*, *CsFUS3*, and *CsSTE11*, respectively. The predicted protein encoded by the *B*. *sorokiniana CsHOG1* homologue consists of 356 amino acids, which is 99% and 90% identical to the *HOG1* in *C*. *heterostrophus* (BAD99295.1, NCBI) and *OSM1* in *M*. *grisea* (AAF09475.1, NCBI), respectively. The predicted protein encoded by the *B*. *sorokiniana CsSLT2* homologue has 436 amino acids, which is 94% and 80% identical to the *MPS1* in *C*. *heterostrophus* (ABM54149.1, NCBI) and *MPS1* in *M*. *grisea* (AAC63682.1, NCBI), respectively. The predicted protein encoded by *CsFUS3* consists of 353 amino acids, which is 99%, 93% and 93% identical to the *CHK1* in *C*. *heterostrophus* (AAF05913.1, NCBI), *PMK1* in *Magnaporthe grisea* (AAC49521.2, NCBI) and *mak-2* in *Neurospora crassa* (AAK25816.1, NCBI), respectively. The predicted protein encoded by *CsSTE11* consists of 960 amino acids, which is 99% and 57% identical to the *STE11* in *C*. *heterostrophus* (BAH97086.1, NCBI) and *nrc-1* in *N*. *crassa* (AAC21676.1, NCBI), respectively.

### Generation of gene knockout mutants for *CsHOG1*, *CsSLT2*, *CsFUS3*, and *CsSTE11*

To investigate the function of these MAPK or MAPKK orthologous genes, we generated knockout mutants for each of them using the split-marker system [17]. The coding region of each gene was replaced by the hygromycin phosphotransferase gene (*hph)* in the knockout mutants as confirmed by Southern hybridization or PCR analysis (Fig 1 and S1–S3 Figs). For *CsHOG1*, three independent transformants were obtained from a knockout experiment. Southern hybridization analysis indicated that a 1.7 kb fragment consisting of most of the coding region of *CsHOG1* present in the wild type strain (ND90Pr) was missing in two of the transformants (∆*Cshog1*-2 and ∆*Cshog1*-4), in which an expected 3.3 kb fragment containing the *hph* was detected (Fig 1). One of the transformants (ect-*Cshog1*-3) had the same 1.7 kb fragment as the wild type (Fig 1), suggesting it is an ectopic transformant. In the two knockout mutants (*∆Csfus3*-1 and *∆Csfus3*-3) obtained for *CsFUS3*, a 9.2 kb fragment in the wild type strain (ND90Pr) was replaced by a 5.9 kb fragment as detected by Southern hybridization using a probe amplified from the 5’ flanking sequence of *CsFUS3* (S1 Fig). For *CsSTE11*, four knockout mutants (*∆Csste11*-1, -2, -3, and -6) were obtained, all of which were missing the 2.2 kb fragment found in the wild type and ectopic transformant (ect-*Csste11*-4) when a probe amplified from the target gene was used for Southern hybridization (S2 Fig). For confirmation of the *CsSLT2* deletion, PCR analysis was performed with the primer CsSLT2-F0 located at the 5’ end flanking region of *CsSLT2* and the primer HY from the *hph* gene. The expected amplicon only appeared in the knockout mutants but not in the wild type (S3 Fig), indicating that *CsSLT2* was replaced by the *hph* gene.

### Developmental and growth phenotypes of gene knockout mutants

**Fig 2:**
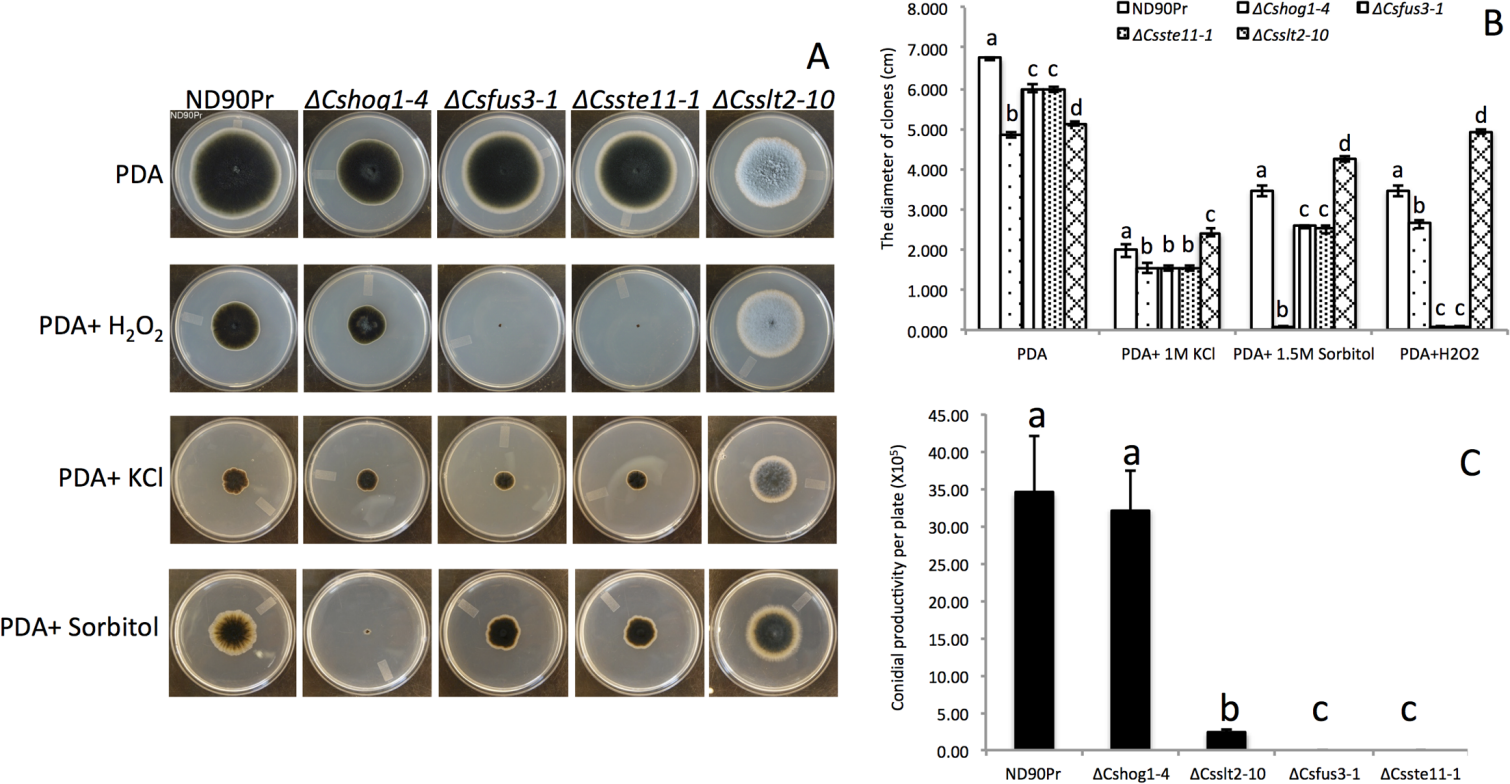
Fungal development and conidial productivity of knockout mutants. A, fungal growth and responses to stresses. Colonies were grown on PDA, PDA with 10mM H_2_O_2_, PDA with 1 M KCl and PDA with 1.5 M sorbitol, respectively, starting with mycelial plugs of uniform size inoculated on the centers of the plates. Photos were taken after growth for 6 days at 25°C. B, Quantitative growth rate of wild-type and mutants grown on PDA, PDA with 1 M KCl, PDA with 1.5 M sorbitol and PDA with 10mM H_2_O_2_, respectively. The colony diameters were measured 6 days after growth on the media. Error bars indicate standard deviation. C, Conidial productivity of wild-type and mutants grown on minimal medium plates for 6 days at 25°C in a cycle of 14 h of light and 10 h of darkness. Error bars indicate standard deviation. Significant differences (P value = 0.001) are indicated by different letters above the columns under each condition.

All mutants were slower in growth on PDA than the wild type (Fig 2A and 2B), with *∆Cshog1* and *∆Csslt2* exhibiting the slowest growth among the four mutants (Fig 2B). The colonies of *∆Csslt2* were morphologically quite different from those of the wild type strain on PDA; they showed less melanization, had more vegetative mycelia and formed less aerial mycelia on PDA than the wild type (Fig 2A). However, no significant differences in colony morphology were observed for the other mutants (*∆Csfus3*, *∆Cshog1* and *∆Csste11*) as compared to the wild type since they all produced black melanized colonies. Nevertheless, conidial productivity of the *∆Csslt2* mutants was significantly reduced, as only 6.77% as many conidia were produced compared with the wild-type strain (Fig 2C), and no conidia were produced by the *∆Csfus3* and *∆Csste11* mutants on minimum media (MM) or V8-PDA (Fig 2C).

When mycelia of *∆Csslt2* mutants were treated with the enzymes (Lysing and Driselase) used for protoplasting, protoplasts were released after 1 hour of incubation at 30°C. In contrast, at least three hours of incubation was required to obtain a similar number of protoplasts for the wild type under the same conditions. This indicated that *CsSLT2* had a role in the maintenance of cell-wall integrity in *B*. *sorokiniana*.

The complemented strains of *∆Csslt2* and *∆Csfus3* mutants had the same morphology as the wild type.

### Responses to different stresses

The growth of wild type and mutants was inhibited by all stress reagents tested in this study. *∆Cshog1* mutants were significantly reduced in growth under the oxidative stress produced by 10mM H_2_O_2_ and the salt stress produced by 1.5mM KCl (Fig 2A and 2B) compared to the wild type, and were completely inhibited by the hyperosmotic stress produced by the 1.5 mM sorbitol (Fig 2A and 2B). The *∆Csfus3* and *∆Csste11* mutants were similar in reactions to all stress conditions tested; both were hypersensitive to the oxidative stress and significantly reduced in growth under hyperosmotic and salt stresses (Fig 2A and 2B). Interestingly, the *∆Csslt2* mutants showed better growth under the different stresses tested in this study compared to the wild type (Fig 2A and 2B), indicating that deletion of *SLT2* lead to mutants with higher tolerance to these stresses.

### Appressorium development

**Fig 3:**
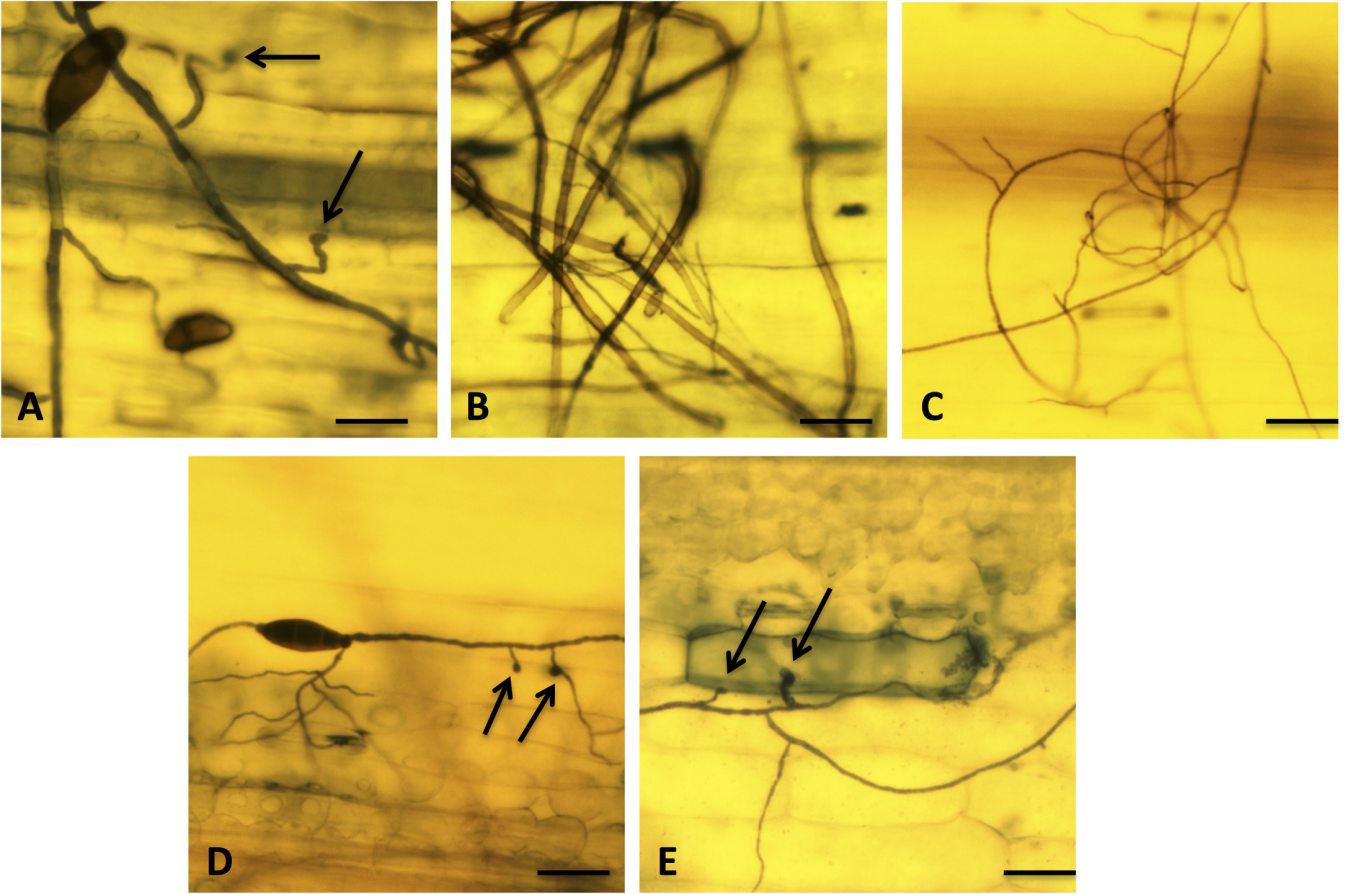
Infection structure differentiation of the wild type (ND90Pr) (A), *∆Csfus3-1* (B), *∆Csste11-1* (C), *∆Cshog1-4* (D) and *∆Csslt2-10* (E) strains at 24 hours after inoculation (HAI) on intact leaves of barley cv. **Bowman.** Fungal and dead plant cells were stained blue with trypan blue. Appressoria are indicated by black arrows. Bars = 60 μm.

Trypan blue staining showed that the *∆Cshog1* and *∆Csslt2* mutants formed normal appressoria-like structures comparable to the wild type strain at 24 HAI on the barley leaves (Fig 3A, 3D, and 3E). However, appressoria formed by *∆Cshog1* and *∆Csslt2* mutants appeared to be smaller than those of the wild type and no appressoria-like structures were observed for the *∆Csfus3* and the *∆Csste11* mutants inoculated on the barley leaves (Fig 3B and 3C).

### Pathogenicity test

**Fig 4:**
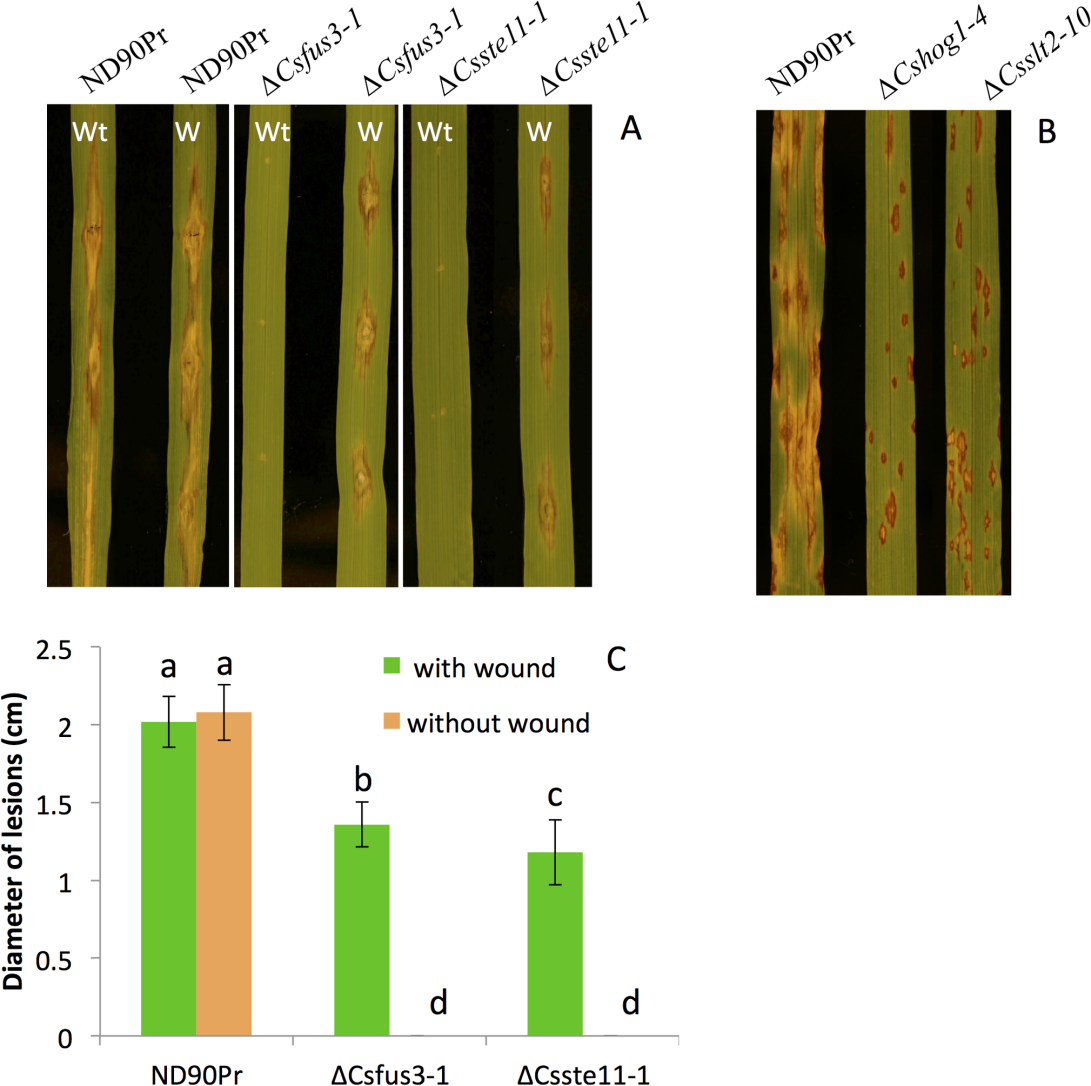
Pathogenicity test of mutants on barley leaves. A, Point-inoculation with mycelial plugs of wild type (ND90Pr), *∆Csfus3-1* and *∆Csste11-1* mutants on non-wounded (Wt) and wounded (W) leaves of barley cv. Bowman. B, Spray inoculation of conidia of wild type (ND90Pr), *∆Cshog1-4* and *∆Csslt2-10* mutants on intact leaves of barley cv. Bowman. C. Quantitative analysis of disease severity based on lesion lengths on inoculated leaves as shown in A. All photographs were taken at 5 days after inoculation (DAI). A syringe needle was used to make wounds on leaves for inoculation with small mycelial plugs (A). Error bars indicate standard deviation. Significant differences (P value = 0.001) are indicated by different letters above the columns.

Since the *∆Csfus3* and *∆Csste11* mutants were unable to produce conidia, small mycelial plugs were used for inoculation. The results showed that both of the mutants lost the ability to penetrate plants and cause the spot blotch disease symptoms when inoculated on unwounded leaves of barley seedling plants (Fig 4A). When inoculated on wounded leaves, both *∆Csfus3* and *∆Csste11* mutants caused infections but the lesion sizes were smaller (~1.3 cm long) in comparison with the wild type, which usually produced lesions larger than 2 cm long (Fig 4A and 4C). The complemented strain for *∆Csfus3* produced normal infection and disease symptoms as the wild type when inoculated on the barley seedling plants (S4 Fig).

When conidia of *∆Cshog1* and *∆Csslt2* mutants were spray inoculated on barley leaves, small necrotic lesions were produced by both of the mutants, but with very little or no chlorosis surrounding the infection sites (Fig 4B). In contrast, large necrotic lesions with extensive chlorosis were caused by the wild type (Fig 4B). This result indicated that these two mutants were significantly reduced in virulence on leaves of barley cv. Bowman. The complemented strain of *∆Csslt2* restored the virulence to the level of the wild type (S4 Fig)

**Fig 5:**
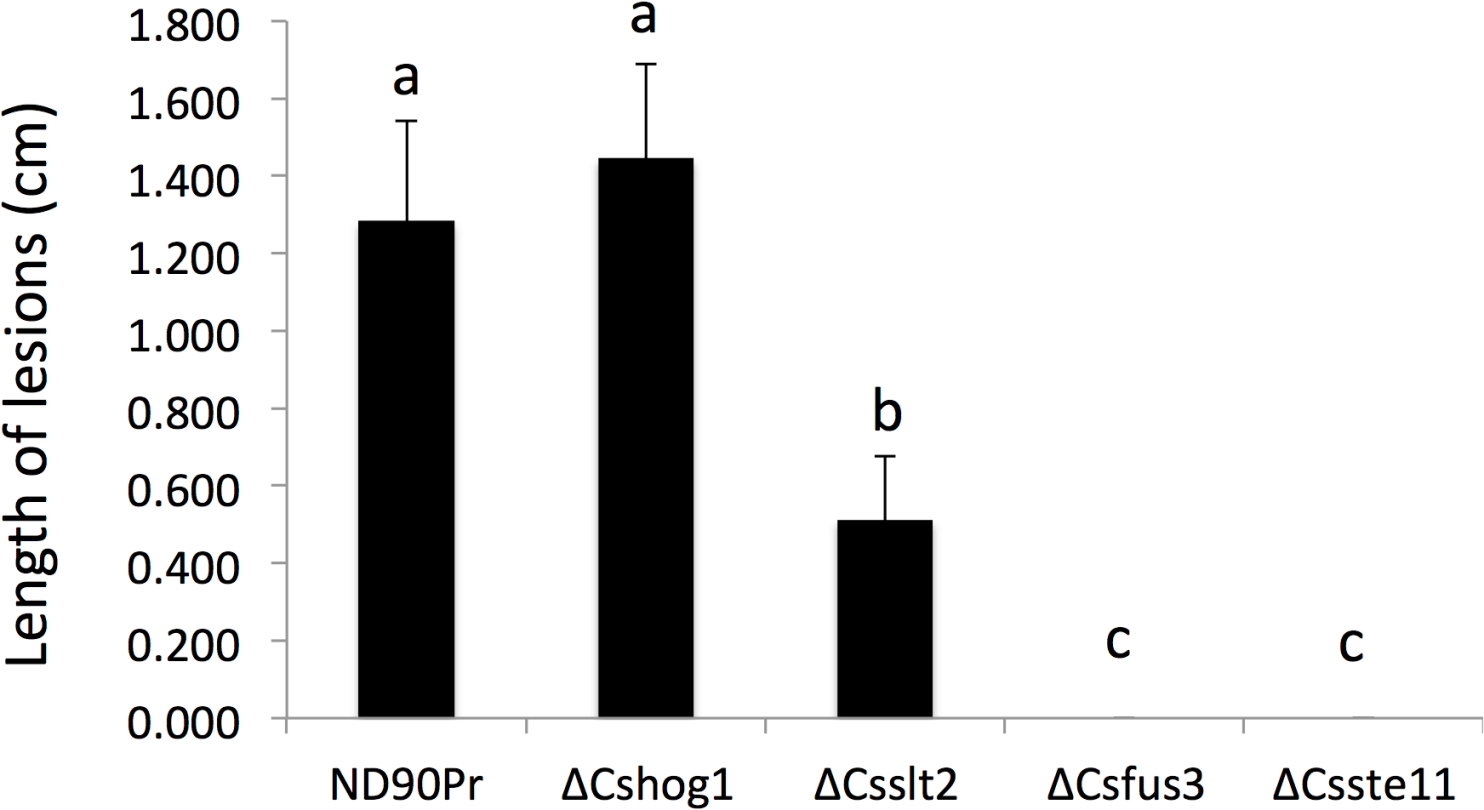
Pathogenicity test on barley roots. Roots (15cm long) of barley seedlings (cv. Bowman) were inoculated with mycelial plugs (2×2 mm^2^) of wild type (ND90Pr) and mutants according to the method described by Liljeroth et al. (23). The length of brown discoloration lesions was measured at 9 days after inoculation. Error bars indicate standard deviation. Significant differences (P value = 0.001) are indicated by different letters above the columns.

*B*. *sorokiniana* also is a pathogen that causes common root rot in barley and wheat. To investigate the effect of deletion of MAPK/MAPKK genes on root infection, we performed inoculation on roots of barley seedling plants. The results showed that both *∆Cshog1* and *∆Csslt2* mutants were able to infect roots and cause discoloration lesions similar to the wild type, but lesions induced by *∆Csslt2* mutants were smaller than those observed for the wild type, while no differences were observed between *∆Cshog1* mutants and wild type on the inoculated barley roots (Fig 5). On barley roots inoculated with *∆Csfus3* and *∆Csste11* mutants, no lesions were observed, indicating that they lost the ability for root infection.

## Discussion

In this study, we have investigated the role of MAPKs involved in three different signaling pathways that have been found in filamentous fungi, including the HOG1 (*h*igh *o*smolarity *g*lycerol according to yeast nomenclature) MAPK pathway, the CWI (*c*ell *w*all *i*ntegrity) MAPK (homologous to Slt2/Mpk1 in yeast) pathway, and the *p*athogenicity *M*AP *k*inase (PMK) pathway (homologous to the mating/filamentation Fus3/Kss1 MAPK pathway of the yeast) [1, 2]. Our results indicated that these MAPKs play similar and unique roles in regulating fungal development and virulence in *B*. *sorokinina* as compared to the orthologues that have previously been characterized in other fungal species.

### The function of *CsHOG1*

In most fungi studied to date, deletion of the *HOG1* orthologs makes the mutants very sensitive to hyperosmotic stress. Examples include *C*. *parasitica*, *B*. *oryzae*, and *M*. *graminicola* [25–27]. In *B*. *oryzae*, the *HOG1* ortholog, *SRM1*, was also found to be involved in resistance to the oxidative stress [26]. A similar phenomenon was found in the human fungal pathogen *Candida albicans*; [28]. In the present study, we demonstrated that *∆Cshog1* mutants were hypersensitive to the hyperosmotic stress produced by 1.5 mM sorbitol and showed slightly reduced growth under oxidative and salt stresses (Fig 2), suggesting that *CsHOG1* shares the same function as those orthologues in other fungi.

In addition to resistance to hyperosmotic stress, *CsHOG1* is also required for full virulence on barley leaves. When conidia of *∆Cshog1* mutants were inoculated on seedlings of cv. Bowman, normal appressoria-like structures formed at 24 HAI (Fig 3D) and necrotic lesions were observed at five DAI, but the ∆*Cshog1* mutants showed reduced virulence on the host compared to the wild type (Fig 4B). This result is consistent with what has been reported for the other two plant pathogens *C*. *heterostrophus* [3] and *C*. *parasitica* [25] and two human pathogens *C*. *albicans* [29] and *Cryptococcus neoformans* [30]. The reduction in virulence of the *∆Cshog1* mutants might be due to their sensitivity to the hyperosmotic stress and reactive oxygen species (ROS), which are produced by the host as a defense response. The breakdown of cell walls by fungal infection might result in high osmotic stress in the host because of the accumulation of glycerol or other compounds [3]. ROS is commonly found in plants, especially during plant infections by pathogens [31]. However, we found no significant difference in virulence on barley seedling roots between *∆Cshog1* mutants and wild type (Fig 5), suggesting that different host defense mechanisms may operate during development of leaf and root diseases, respectively.

The HOG1 signaling pathway was found to be required for other fungal development and growth in some other fungi. In *C*. *parasitica*, the *∆cpmk1* mutants showed reduced pigmentation and conidiation [25]. In *Botrytis cinerea*, *BcSak1* was found to be involved in the formation of conidia and appressoria-like structures as well [32]. In the human pathogen *C*. *albicans*, the *CaHog1* was even required for cell wall biosynthesis [29]. However, we did not observe such phenotype changes in the *∆Cshog1* mutants of *B*. *sorokiniana* in the present study. All the studies mentioned above further indicate that the functions of MAP kinase genes might be different among fungi, although the signal transducers are highly conserved among them.

### The function of *CsSLT2*

In *S*. *cerevisiae*, the MAP kinase gene *SLT2* was found to be involved in the cell integrity pathway, which monitors cell wall integrity and cell wall biosynthesis [2, 33]. It was also found to be involved in the transduction of environmental signals such as low osmolarity, high temperature and nutrient limitations [2]. In this study, we showed that *∆Csslt2* mutants were more sensitive to the cell wall degrading enzymes and had reduced production of conidia and melanin, suggesting that *CsSLT2* play an important role in some normal fungal development processes including cell wall integrity, asexual reproduction and biosynthesis of melanin. Similar functions of *SLT2* have been reported in some other plant pathogenic fungi such as *C*. *heterostrophus* [3], *M*. *grisea* [9] and *Claviceps purpurea* [34]. In *Fusarium graminearum*, Hou et al. [35] demonstrated that the orthologue of *SLT2* gene (*Mgv1*) is involved in sexual reproduction, plant infection, and cell wall integrity, but it is not required for conidiation. However, the *SLT2* orthologues were not involved in the biosynthesis of melanin in all fungi mentioned above except *C*. *heterostrophus* [36] and *B*. *sorokiniana* in this study. When the conidia of *∆Csslt2* mutants of *B*. *sorokiniana* were inoculated on the barley plants, the normal appressoria-like structures were found 24 hours after inoculation (Fig 3E) and the mutants were pathogenic but reduced in virulence compared to the wild type (Fig 4B). Also, the *∆Csslt2* mutants showed reduced virulence when inoculated on barley root (Fig 5). These results suggest that *CsSLT2* is not involved in the formation of functional appressoria but required for full virulence on barley plants. The reduction of virulence could be due to the defects in the cell wall integrity of *∆Csslt2* mutants, which allowed cell walls of the mutants to be easily degraded by the cell wall degrading enzymes produced by the host. The same phenomenon was found in some other fungi including *C*. *heterostrophus* [3] and *M*. *graminicola* [37]. However, in *M*. *grisea*, the *MPS1* gene was required for functional appressoria formation and host penetration, and thus *∆mps1* mutants are nonpathogenic [9]. In *C*. *purpurea*, the *Cpmk2* gene was also required for penetration since the *∆Cpmk2* mutants only had limited ability to colonize and grow invasively in the host [34]. In *F*. *graminearum*, *Mgv1* is not involved in pathogenecity but is related to deoxynivalenol accumulation [35]. Interestingly, the *∆Csslt2* mutants of *B*. *sorokiniana* showed improved resistance to all stresses tested in this study, including oxidative stress as well as hyperosmotic and salt stresses, as compared to the wild type. Similar phenomena were reported in the related fungus, *C*. *heterostrophus* [3]. The reason for this improved resistance in the *∆Csslt2* mutants to the different stresses is still unknown. It is possible that the SLT2 signaling pathway negatively regulates the production of some compounds which are required for resistance to the stresses and loss of *SLT2* leads to the overproduction of those compounds, resulting in improved resistance in the mutants.

### The function of *CsFUS3*

The *FUS3/KSS1* MAP kinase pathway is the best characterized of the three MAP kinase pathways in fungi [2]. In *S*. *cerevisiae*, the *FUS3/KSS1* MAP kinase pathway was essential for regulating the mating process and filamentous growth, however, in filamentous fungi, it has more functions than in *S*. *cerevisiae*, including appressorium formation, conidiation, spore germination, infectious growth, pathogenicity and virulence [2]. In this study, we showed that *CsFUS3* was required for appressorium formation, conidiation and resistance to oxidative stress. In all appressorium-forming pathogens studied so far, homologs of *FUS3/KSS1* are involved in the formation of functional appressoria. Examples include *M*. *grisea* [7], *C*. *heterostrophus* [3], *Colletotrichum lagenarium* [38], *Pyrenophora teres* [39] and *B*. *sorokiniana* in this study. Without functional appressoria, all *∆Csfus3* mutants were non-pathogenic on both healthy leaves and roots of barley. The *FUS3/KSS1* homologs were also found to be essential for conidiation in some other fungi, including *C*. *heterostrophus* [3], *P*. *teres* [39], *F*. *graminearum* [40], *B*. *oryzae* [41], *Stagonospora nodorum* [42] and *C*. *parasitica* [43]. Inoculation on wounded leaves showed that the *∆Csfus3* mutants were able to infect the host through the wounded sites but showed reduced virulence compared to the wild type (Fig 4A and 4C), indicating that the FUS3 signaling pathway is also required for infectious hyphal growth after penetration in *B*. *sorokiniana*. This result is similar to what has been reported in some other fungi, including *M*. *grisea* [7] and *C*. *heterostrophus* [5]. The reduced virulence might also be due to the hypersensitivity of the *∆Csfus3* mutants to the oxidative stress because the ROS is one of the important host defense responses [31].

In most fungi, homologs of *FUS3/KSS1* are not involved in regulating the response to H_2_O_2_ stress [2], but hyposensitivity of the mutants to the oxidative stress has been found in *C*. *heterostrophus* [6] and *B*. *sorokiniana* (this study). This indicates that the adaptation to the oxidative stress in these two closely related fungal species is mainly regulated via the FUS3-type MAP kinase pathway instead of the HOG1-type pathway. In some other fungi, disruption of the FUS3 signaling pathway led to reduced virulence but the mutants were still pathogenic. For example, in *S*. *nodorum*, ∆*mak2* mutants could infect the wheat plant host through natural openings but showed reduced virulence by causing only limited necrosis on leaves [42]. A similar phenomenon was also found in *C*. *parasitica* where the *∆cpmk2* mutants showed reduced growth rate and smaller canker size on the host [43]. In addition to these functions mentioned above, the FUS3 signaling pathway was also found to be involved in other fungal development. For example, in *C*. *lagenarium*, *Cmk1* is not only involved in appressorium formation and pathogenicity, but also affects conidial germination [38]. In most fungi, the homologs of *FUS3/KSS1* are not involved in the biosynthesis of melanin, except in *C*. *heterostrophus* [6, 36] and *M*. *graminicola* [44].

### The function of *CsSTE11*

In *S*. *cerevisiae*, *STE11* is a MAPKK kinase gene in the upstream of *FUS3/KSS1* and *HOG1* pathways which regulate the mating response and filamentous growth and the resistance to the hyperosmotic and other stress response, respectively [2]. In this study, the ∆*Csste11* mutants shared all the phenotypes of the ∆*Csfus3* mutants, including defects in conidiation and formation of appressoria-like structures, hypersensitivity to the oxidative stress, and reduction in virulence. However, the ∆*Cshog1* and ∆*Csste11* mutants were different in most of the phenotypes when compared to ∆*Csfus3* and ∆*Csste11* mutants. In filamentous fungi, the upstream regulator of the FUS3 MAP kinase pathway has not been well characterized. Our results indicated that Cs*STE11* only regulates the FUS3 MAP kinase pathway but does not affect the HOG1 MAP kinase pathway in *B*. *sorokiniana*. This result is consistent with those reported in two other fungi, *M*. *grisea* [1] and *C*. *heterostrophus* [6]. In *M*. *grisea*, Zhao et al. [1] found that *MST11*, the ortholog of *STE11*, regulates the MAP kinase gene *PMK1*, which is involved in appressorium formation and invasive growth. In *C*. *heterostrophus*, Izumitsu et al. [6] found that both *STE11* and *CHK1* genes had similar functions in the conidiation, sexual development, melanization, resistance to the hyperosmotic and oxidative stresses and appressorium formation.

In summary, all of three MAP kinase pathways were identified in *B*. *sorokiniana* and contributed to the regulation of fungal development under normal and stress conditions; they are also required for the full virulence of *B*. *sorokiniana* on the barley plants. The MEKK kinase gene *STE11* only regulated the FUS3 pathway, which is required for conidiation, appressorium formation and resistance to oxidative stress. The SLT2 signaling pathway regulated the cell wall integrity, asexual reproduction and biosynthesis of melanin. The HOG1 signaling pathway is required for the resistance to the hyperosmotic stress. This study provides important information on the role of MAP kinase pathways in regulating fungal development and virulence of *B*. *sorokiniana* in the barley plant host.

## Supporting Information

S1 Fig Generation of *∆Csfus3* strains of *Bipolaris sorokiniana*. A, a diagram showing replacement of the *CsFUS3* gene by a 2.6 kb fragment carrying the *E*. *coli* hygromycin phosphotransferase gene (*hph*) using the split-marker system [17]. B, Southern hybridization of *Xba* I-digested genomic DNA from the wild type and *∆Csfus3* strains using probe amplified with primers CsFUS3-F1+CsFUS3-F2. The 9.2 kb fragment in the wild type strain (ND90Pr) was replaced by the 5.9 kb fragment in the *∆Csfus3* strains (*∆Csfus3*-1 and *∆Csfus3*-3).
(TIF)

S2 Fig Generation of *∆Csste11* strains of *Bipolaris sorokiniana*. A, a diagram showing replacement of the Cs*STE11* gene by a 2.6 kb fragment carrying the *E*. *coli* hygromycin phosphotransferase gene (*hph*) using the split-marker system [17]. B, Southern hybridization of *EcoR* V-digested genomic DNA from the wild type and *∆Csste11* strains using probe amplified with primers Cs*STE11*-F5+Cs*STE11*-F6 within the Cs*STE11* gene. The 2.1 kb fragment in the wild type strain (ND90Pr) was not detected in the *∆Csste11* strains (*∆Csste11*-1, -2, -3, and -6). *ect-Csste11*-4 is an ectopic transformant.
(TIF)

S3 Fig Generation of *∆Csslt2* strains of *Bipolaris sorokiniana*. A, a diagram showing replacement of the *CsSLT2* gene by a 2.6 kb fragment carrying the *E*. *coli* hygromycin phosphotransferase gene (*hph*) using the split-marker system [17]. B. Confirmation of *∆Csslt2* mutants by PCR analysis using primers F0 and HY indicated in A. A 2.8kb fragment was only amplified from the *∆Csslt2* strains (*∆Csslt2-5*, *-8*, *-9*, *-10*).
(TIF)

S4 Fig Pathogenicity test of complemented strains for *∆Csfus3-1* and *∆Csslt2* on barley cv. **Bowman leaves.** Conidia of wild type (ND90Pr), *∆Csfus3-1+FUS3-*1, *∆Csslt2* and *∆Csslt2-10+CsSLT2-1* were spray inoculated on leaves of barley cv. Bowman. Disease ratings and photography were conducted at 6 days after inoculation (DAI).
(TIF)
