# Supplementary figures and images for: The Role of Mitogen-Activated Protein (MAP) Kinase Signaling Components in the Fungal Development, Stress Response and Virulence of the Fungal Cereal Pathogen Bipolaris sorokiniana
Source: PLoS One. 2015 May 26;10(5):e0128291. doi: 10.1371/journal.pone.0128291 (PMC4443973; doi:10.1371/journal.pone.0128291)

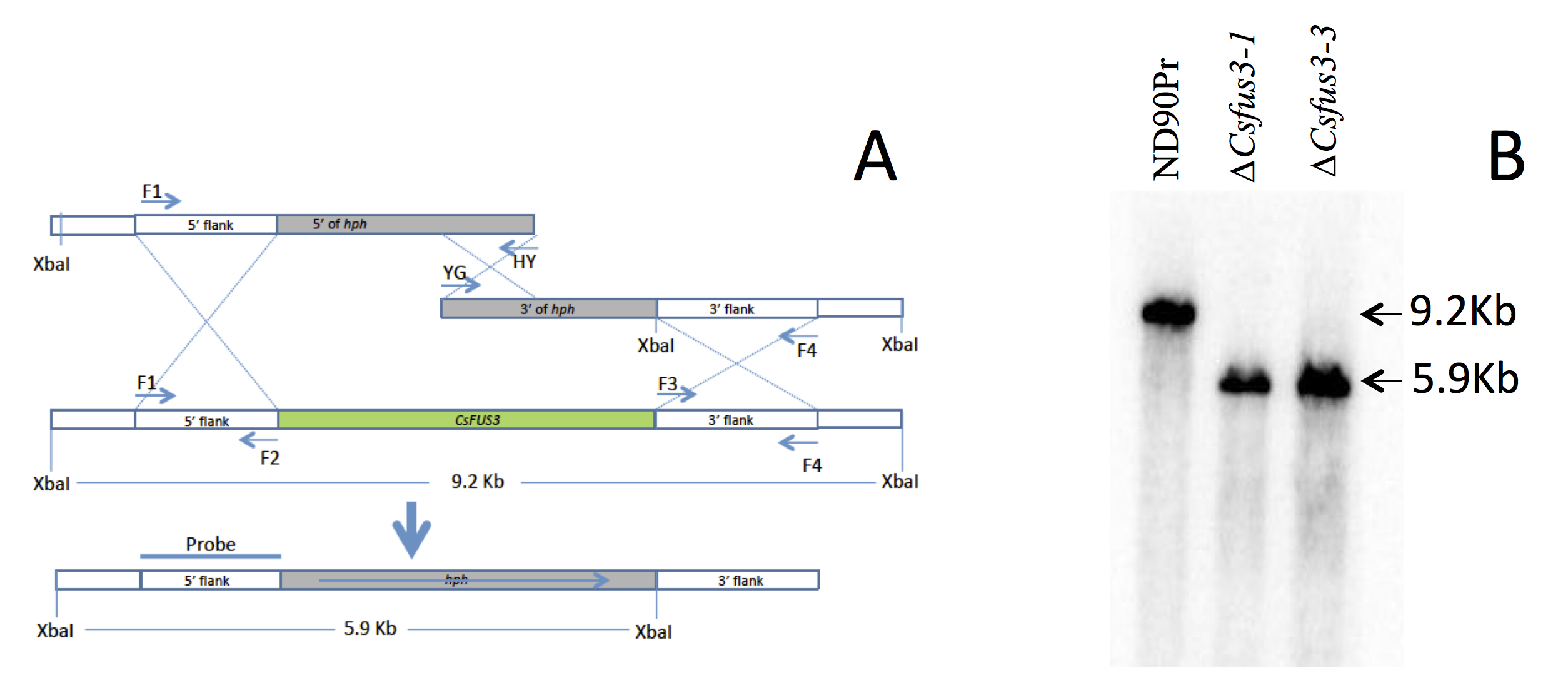

Supplement: S1 Fig — A, a diagram showing replacement of the CsFUS3 gene by a 2.6 kb fragment carrying the E. coli hygromycin phosphotransferase gene (hph) using the split-marker system [17]. B, Southern hybridization of Xba I-digested genomic DNA from the wild type and ∆Csfus3 strains using probe amplified with primers CsFUS3-F1+CsFUS3-F2. The 9.2 kb fragment in the wild type strain (ND90Pr) was replaced by the 5.9 kb fragment in the ∆Csfus3 strains (∆Csfus3-1 and ∆Csfus3-3). (TIF) [file pone.0128291.s001.tif]

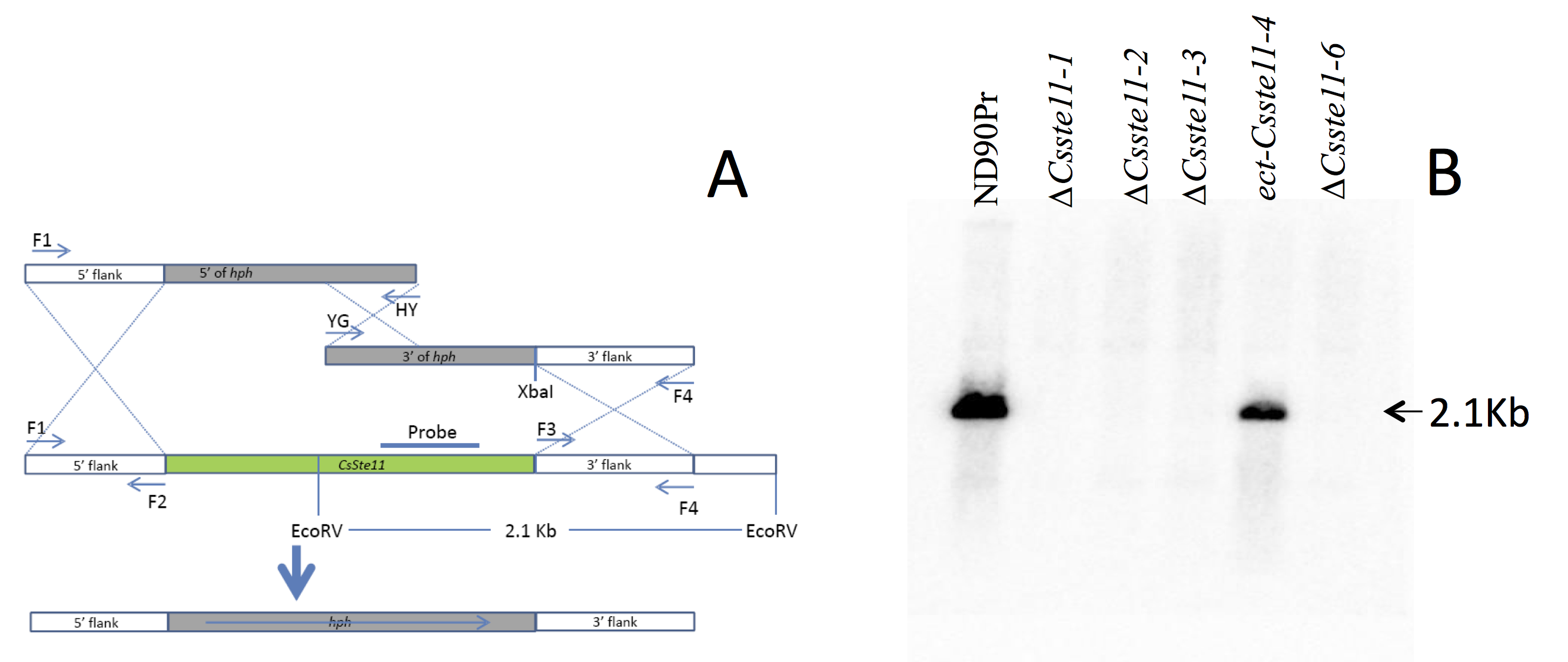

Supplement: S2 Fig — A, a diagram showing replacement of the CsSTE11 gene by a 2.6 kb fragment carrying the E. coli hygromycin phosphotransferase gene (hph) using the split-marker system [17]. B, Southern hybridization of EcoR V-digested genomic DNA from the wild type and ∆Csste11 strains using probe amplified with primers CsSTE11-F5+CsSTE11-F6 within the CsSTE11 gene. The 2.1 kb fragment in the wild type strain (ND90Pr) was not detected in the ∆Csste11 strains (∆Csste11-1, -2, -3, and -6). ect-Csste11-4 is an ectopic transformant. (TIF) [file pone.0128291.s002.tif]

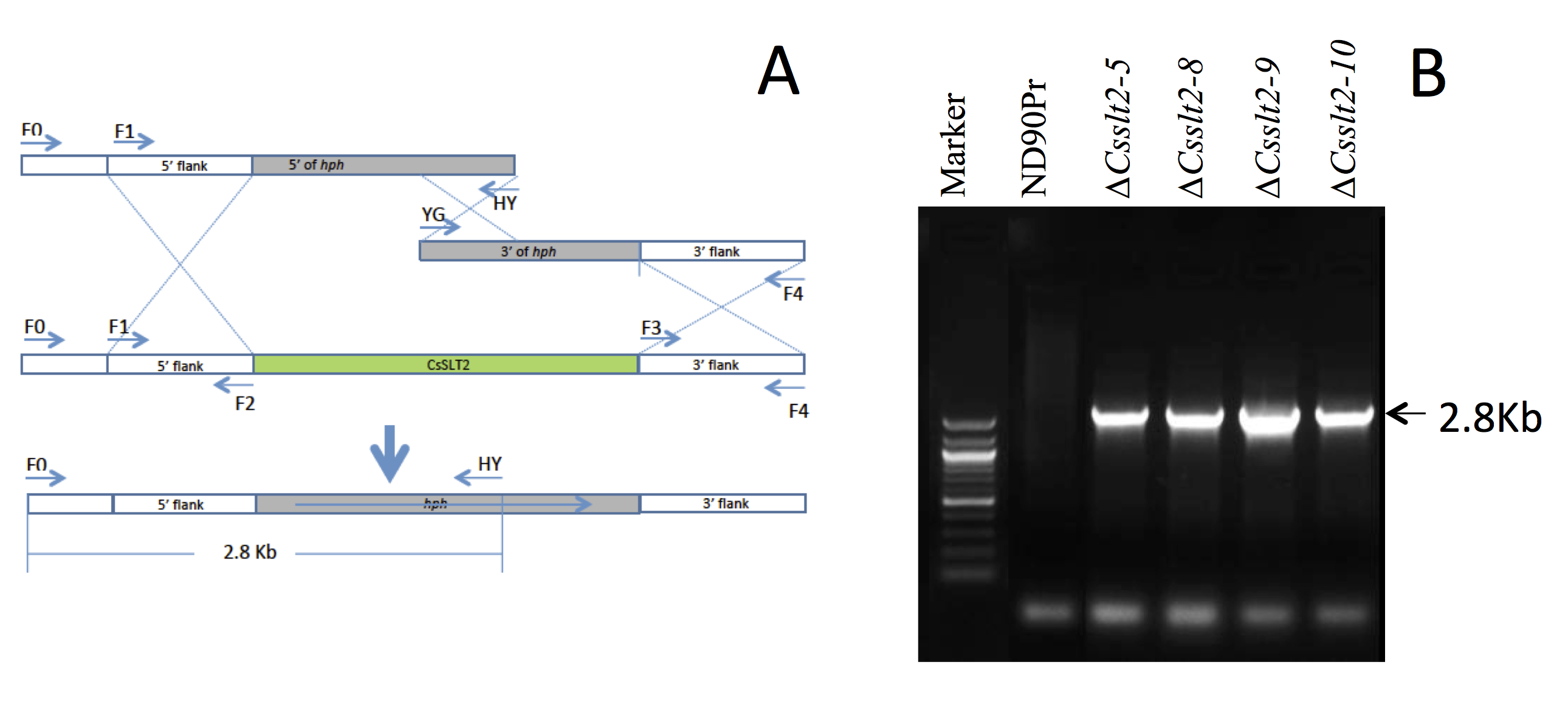

Supplement: S3 Fig — A, a diagram showing replacement of the CsSLT2 gene by a 2.6 kb fragment carrying the E. coli hygromycin phosphotransferase gene (hph) using the split-marker system [17]. B. Confirmation of ∆Csslt2 mutants by PCR analysis using primers F0 and HY indicated in A. A 2.8kb fragment was only amplified from the ∆Csslt2 strains (∆Csslt2-5, -8, -9, -10). (TIF) [file pone.0128291.s003.tif]

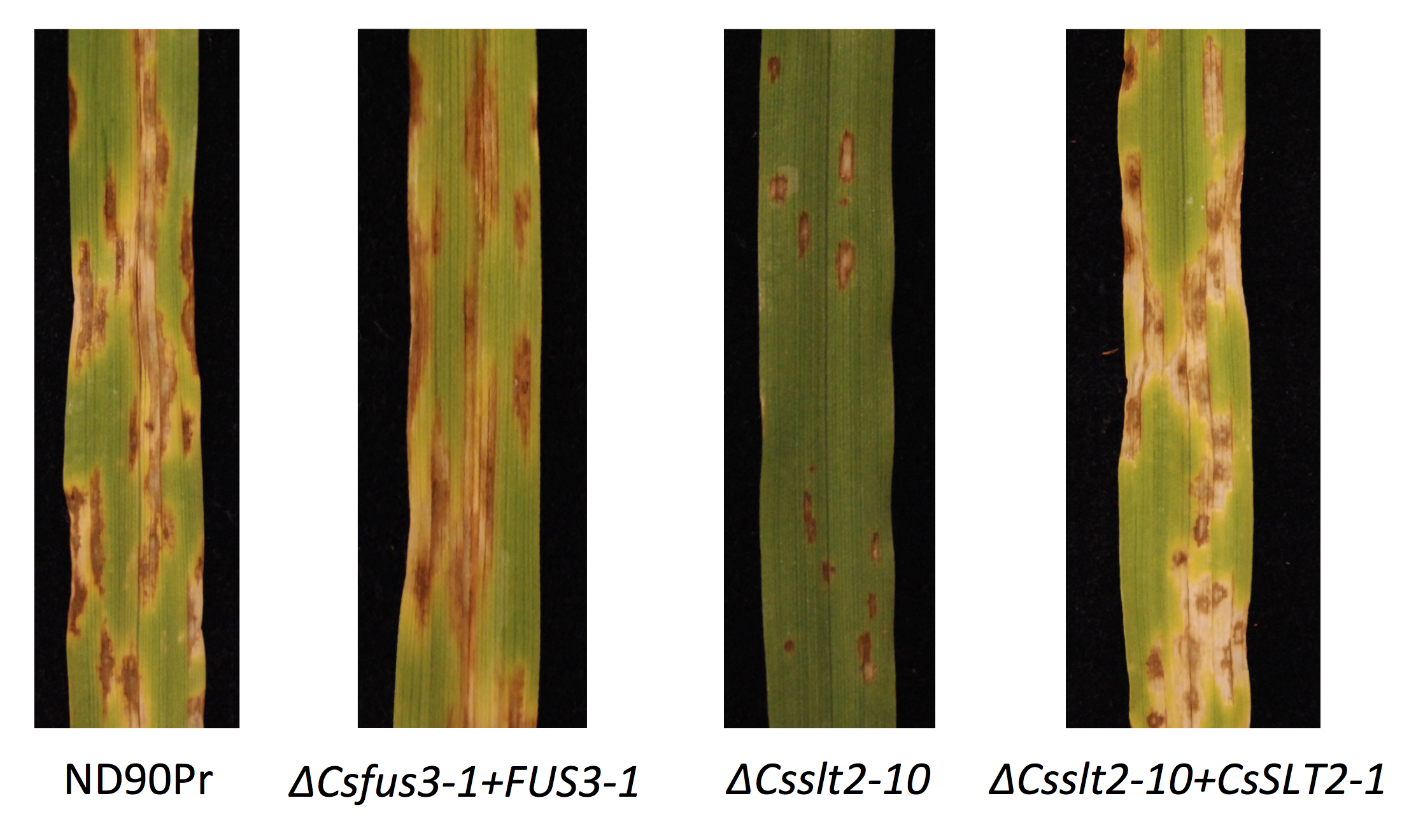

Supplement: S4 Fig — Bowman leaves. Conidia of wild type (ND90Pr), ∆Csfus3-1+FUS3-1, ∆Csslt2 and ∆Csslt2-10+CsSLT2-1 were spray inoculated on leaves of barley cv. Bowman. Disease ratings and photography were conducted at 6 days after inoculation (DAI). (TIF) [file pone.0128291.s004.tif]
